# Phosphorylation of K^+^ channels at single residues regulates memory formation

**DOI:** 10.1101/lm.040816.115

**Published:** 2016-04

**Authors:** Jeffrey Vernon, Elaine E. Irvine, Marco Peters, Jeshmi Jeyabalan, K. Peter Giese

**Affiliations:** 1 Wolfson Institute for Biomedical Research, University College London, London WC1E 6BT, United Kingdom; 2 MRC Clinical Sciences Centre, Imperial College London, Hammersmith Campus, London W12 ONN, United Kingdom; 3 Dart Neuroscience, 12278 Scripps Summit Drive, San Diego, California 92131, USA; 4 Centre for the Cellular Basis of Behaviour, Institute of Psychiatry, Psychology and Neuroscience, King's College London, London SE5 9NU, United Kingdom

## Abstract

Phosphorylation is a ubiquitous post-translational modification of proteins, and a known physiological regulator of K^+^ channel function. Phosphorylation of K^+^ channels by kinases has long been presumed to regulate neuronal processing and behavior. Although circumstantial evidence has accumulated from behavioral studies of vertebrates and invertebrates, the contribution to memory of single phosphorylation sites on K^+^ channels has never been reported. We have used gene targeting in mice to inactivate protein kinase A substrate residues in the fast-inactivating subunit K_v_4.2 (T38A mutants), and in the small-conductance Ca^2+^-activated subunit SK1 (S105A mutants). Both manipulations perturbed a specific form of memory, leaving others intact. T38A mutants had enhanced spatial memory for at least 4 wk after training, whereas performance in three tests of fear memory was unaffected. S105A mutants were impaired in passive avoidance memory, sparing fear, and spatial memory. Together with recent findings that excitability governs the participation of neurons in a memory circuit, this result suggests that the memory type supported by neurons may depend critically on the phosphorylation of specific K^+^ channels at single residues.

Classic studies on conditioning in the invertebrate *Hermissenda crassicornis* suggested that phosphorylation of K^+^ channels acts as a switch for associative memory formation (Alkon 1984). Phosphorylation of K^+^ channels by kinases on serine, threonine, tyrosine, or histidine side chains may regulate memory formation also in mammals, since phosphorylation affects neurotransmitter release, the probability of synaptic transmission, integration by neurons of their dendritic inputs, and neuronal firing (Giese et al. 2001; Zhang and Linden 2003; Zhou et al. 2009; Oh and Disterhoft 2014; Yiu et al. 2014). A direct test of the hypothesis that K^+^ channel phosphorylation regulates memory formation was lacking, as no specific inactivation of a single phosphorylation site in a K^+^ channel subunit in a behaving animal was attempted.

K^+^ channels show great diversity in the mammalian nervous system, presumably enabling fine-tuning of neuronal excitability (Pongs 2008; Jan and Jan 2012). Among these, the voltage-gated K^+^ channel subunit K_v_4.2 has evoked much interest, since its biophysical properties may explain how neurons implement learning at a cellular level in the hippocampus. K_v_4.2 mediates transient K^+^ currents in dendrites of hippocampal CA1 pyramidal neurons (Chen et al. 2006), which regulate the back-propagation of action potentials from the neuron soma into the dendritic tree (Hoffman et al. 1997), and may therefore permit the coincidence of signals necessary to some models of synaptic strengthening (Waters et al. 2005). Additionally, internalization of K_v_4.2 at synaptic membranes accompanies long-term potentiation (Kim et al. 2007), a likely memory mechanism (Giese 2012). K_v_4.2 current levels regulate the subunit composition of synaptic NMDA receptors, thereby controlling the degree of synaptic strengthening (Jung et al. 2008). K_v_4.2 can be phosphorylated on various side chains by different protein kinases (Varga et al. 2000). Some of these phosphorylations have been proposed to regulate K_v_4.2 function for example via control of back-propagation and subcellular distribution (Yuan et al. 2002). Complete deletion of K_v_4.2 in mice affects hippocampus-dependent memory formation (Lugo et al. 2012), but a direct role for K_v_4.2 phosphorylation in learning and memory has not been established.

Small-conductance Ca^2+^-activated K^+^ (SK) channels also regulate excitability in hippocampal CA1 pyramidal neurons. The SK2 subunit contributes to a post-spike decrease of membrane excitability, the medium after-hyperpolarization (AHP), which thereby regulates neuronal firing rates (Bond et al. 2004). Consistent with its effect on dampening the membrane response, SK2 overexpression impairs memory formation (Lugo et al. 2012). Hippocampal CA1 pyramidal neurons express the SK1 subunit in comparable amounts to SK2 (Hammond et al. 2006), but studies in vitro have been hampered by the reported difficulty of expressing the SK1 subunit from rodents, the apparent absence of a homomeric SK1 channel, and pharmacological differences between rodent and human channels (Stocker and Pedarzani 2000; Nolting et al. 2007). Complete deletion of SK1 in mutant mice does not affect the AHP in mouse CA1 pyramidal neurons (Bond et al. 2004), but recent evidence implies that SK1 in some cells is required to maintain an AHP current when ATP levels fall, and that it may therefore act as a current modulator that depends on the metabolic state of the cell (Andres 2012). Protein kinase A (PKA) modulation of the AHP in CA1 pyramidal neurons has been detected during memory formation (Pedarzani et al. 1998; Oh and Disterhoft 2014), but it is unknown whether SK1 or its PKA phosphorylation is functional in learning and memory.

To directly test the proposition that K^+^ channel phosphorylation regulates memory formation we have carried out gene targeting in mice to inactivate a single PKA phosphorylation site in the two K^+^ channel subunits, SK1 and K_v_4.2. Loss of a PKA site either enhanced or impaired a specific type of memory, leaving other memories intact. Thus, K^+^ channel phosphorylation at a single residue regulates formation of a specific memory.

## Results

### Loss of PKA phosphorylation of K_v_4.2 at threonine-38 enhances spatial memory

**Figure 1.**
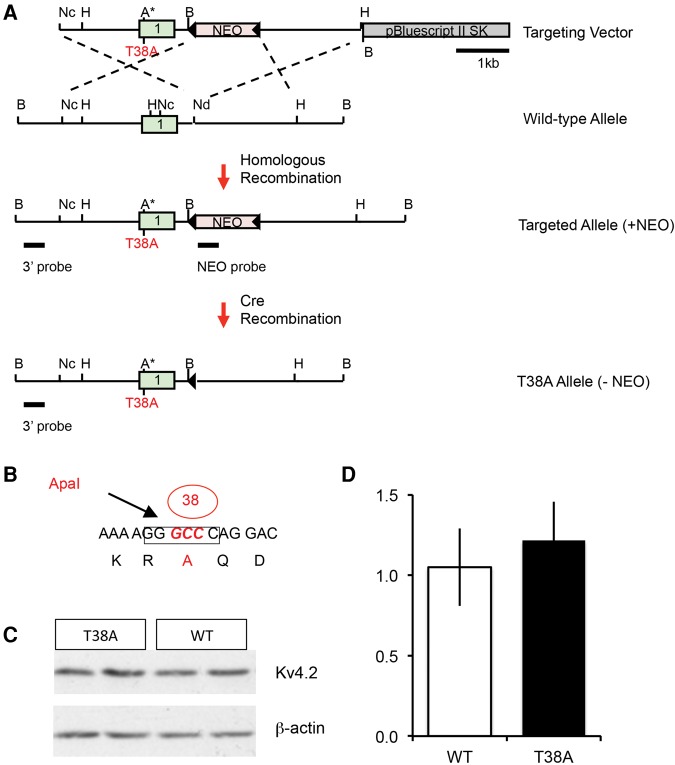
Generation of mice with a T38A point mutation in the K_v_4.2 gene. This mutation prevents PKA phosphorylation of K_v_4.2 at threonine-38. (*A*,*B*) The T38 encoding sequence of the K_v_4.2 gene was mutated to an alanine-coding sequence, resulting also in the introduction of a diagnostic ApaI site for detection of the point mutation. The T38A point mutation was introduced into the K_v_4.2 gene in ES cells. The NEO gene was removed by Cre recombination in resulting heterozygous mutants. Primers Kv4.2seq12s and Kv4.2seq13a were used for genotyping. (A) ApaI; (B) BamHI; (H) HindIII; (Nc) NcoI; (Nd) NdeI. Triangles indicate loxP sites. (*C*,*D*) Western blot analysis indicated that the loxP site in the intron does not affect overall K_v_4.2 expression in hippocampus of homozygous T38A mutants. Panel *C* shows a representative result for two T38A mutants and two WT littermates. Panel *D* shows the result of quantification of K_v_4.2 expression normalized to β-actin levels (mean ± sem; *n* = 3 per genotype; *t*-test: *P* = 0.61).

**Figure 2.**
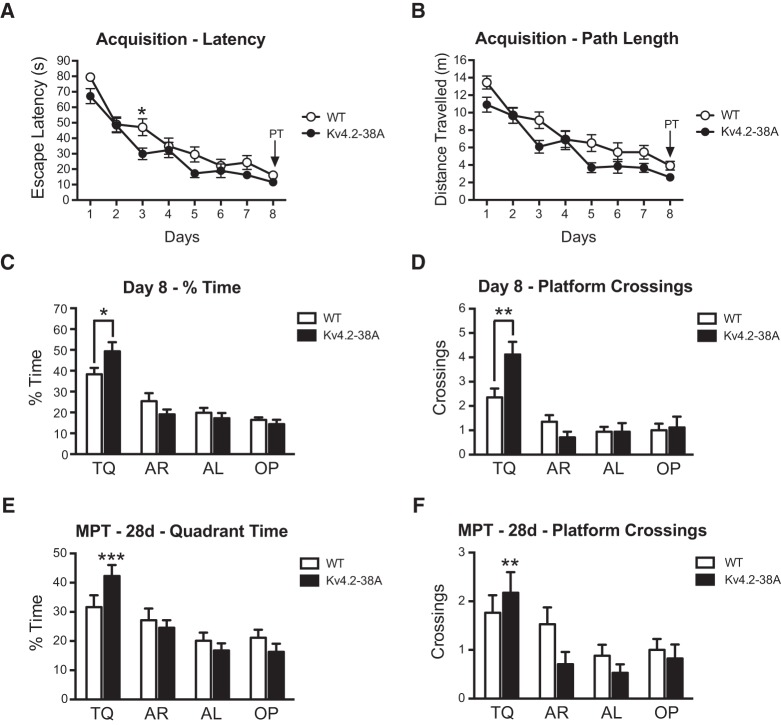
Loss of PKA phosphorylation of K_v_4.2 at threonine-38 enhances spatial memory formation. (*A*,*B*) T38A mice (*n* = 17) were improved in their ability to locate the platform during training in the Morris water maze in comparison with their WT littermates (*n* = 17). *A* probe trial (PT) was given at the end of training and a memory probe trial (MPT) 28 d after training. (*C*,*D*) The probe trial given at the end of training showed that the T38A mice had enhanced spatial memory as they spent significantly more time searching in the TQ than their WT littermates and they crossed the exact platform location more frequently. During this probe trial WT mice did swim faster than T38A mutants (WT: 26.1 ± 0.6 cm/sec; T38A: 23.8 ± 0.7 cm/sec; *P* < 0.05). (*E*,*F*) During the memory probe trial 28 d after training the T38A mice spent more time in the target quadrant (TQ), whereas the WT mice had forgotten the location of the platform and searched randomly. The T38A mice also made significantly more crossings over the previous platform location, whereas no significant selectivity was observed for the WTs. During this memory probe trial WT mice did swim with a similar average speed as T38A mutants (WT: 24.3 ± 0.9 cm/sec; T38A: 23.0 ± 0.7 cm/sec; *P* = 0.44). TQ, target quadrant, AR, adjacent right quadrant; AL, adjacent left quadrant; OP, opposite quadrant. Mean ± SEM. (*)*P* < 0.05; (**)*P* < 0.01; (***)*P* < 0.001 compared with all other quadrants.

K_v_4.2 contains 630 amino acid residues, and is phosphorylated by PKA at both threonine-38 (T38) and serine-552 (S552) (Varga et al. 2000). We generated mutant mice with a knock-in mutation (T38A), which by substituting alanine for the native threonine specifically blocks phosphorylation at T38 (Fig. 1), a residue that is conserved between mouse, chimp, and human (not shown). K_v_4.2^T38A/T38A^ (T38A) mice were tested for hippocampus-dependent spatial reference memory formation, using the hidden-platform version of the Morris water maze (Fig. 2; Morris et al. 1982). Mice were trained with four trials per day for 8 d. Two-way repeated-measures ANOVA showed that both genotypes improved their performance over the course of the training period, but T38A mice showed significantly improved acquisition compared with their wild-type (WT) littermates (effect of genotype for latency, *F*_(1,32)_ = 5.0, *P* < 0.05; effect of genotype for path length, *F*_(1,32)_ = 7.6, *P* < 0.01; Fig. 2A,B). However, task acquisition is not a good measure of spatial memory formation, as mice may succeed in locating the platform using nonspatial strategies. In order to distinguish these alternatives a probe trial was given immediately after training (Fig. 2C,D). Both genotypes formed a spatial memory as both groups spent significantly more time in the target quadrant compared with all other quadrants (WT, *F*_(3,64)_ = 11.7, *P* < 0.001; T38A, *F*_(3,64)_ = 30.3; *P* < 0.001), but this was enhanced in T38A mice, which spent significantly more time in the target quadrant than WT mice (WT, 38.3% ± 3.1%; T38A, 49.3% ± 4.4%; *t* = 2.1, two-tailed; *P* < 0.05; Fig. 2C). Moreover, they made more crossings of the exact platform location in comparison to controls (WT, 2.4 ± 0.4; T38A, 4.1 ± 0.5; *t* = 2.8, two-tailed; *P* < 0.01; Fig. 2D). Additionally, mice were tested for remote reference memory 28 d after the last training session in the pool. T38A mice spent significantly more time in the target quadrant compared with all other quadrants (*F*_(3,64)_ = 16.9, *P* < 0.001) and made more crossings over the platform location (*F*_(3,64)_ = 6.4, *P* < 0.001), whereas WT mice searched randomly 28 d after the end of training (% Time, *F*_(3,64)_ = 2.4, not significant [NS]; platform crossings, *F*_(3,64)_ = 2.4, NS; Fig. 2E,F). Thus, inhibition of phosphorylation of K_v_4.2 at T38 enhances both recent and remote spatial reference memory.

### Loss of threonine-38 phosphorylation of K_v_4.2 does not affect memory after fear conditioning or passive avoidance training

**Figure 3.**
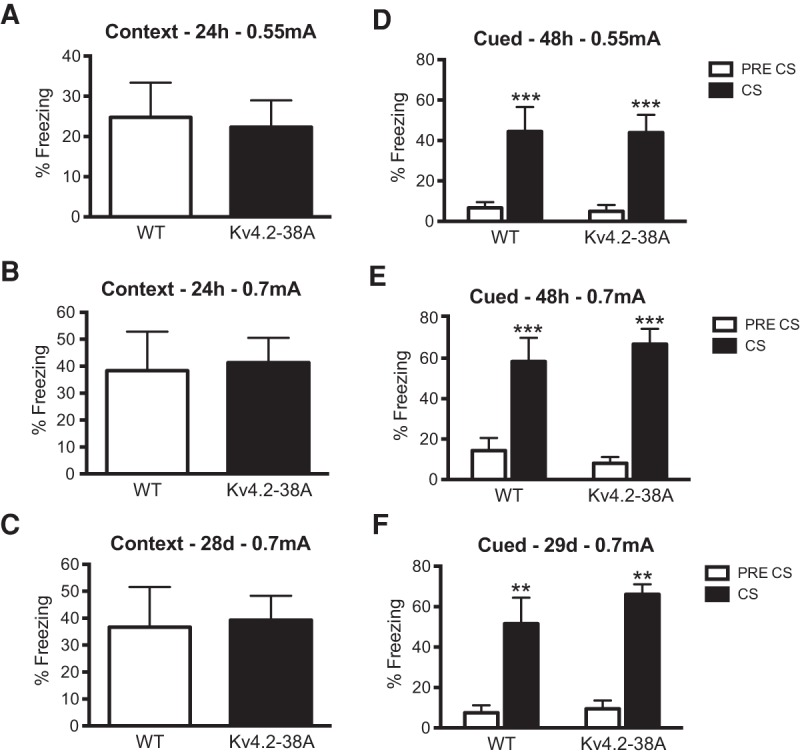
Loss of PKA phosphorylation of K_v_4.2 at threonine-38 does not affect contextual and cued fear memory formation. (*A*) Mice were trained in a weak fear conditioning paradigm with one tone/shock pairing (0.55 mA, 80 dB). Contextual fear memory was normal in T38A mice (*n* = 5) 24 h after training as freezing levels did not differ from WT mice (*n* = 7). (*B*) Mice were trained in fear conditioning with one tone/shock pairing (0.7 mA, 80 dB). Contextual fear memory was normal in T38A mice (*n* = 10) 24 h and 28 d (*C*) after training as freezing levels did not differ from WT mice (*n* = 7). (*D*) T38A mice had normal cued fear memory when tested 48 h after training with low shock intensity (0.55 mA). (*E*) T38A mice had normal cued fear memory when tested 48 h or 29 d (*F*) after training with high shock intensity (0.7 mA). Mean ± SEM. (**)*P* < 0.01; (***)*P* < 0.001 compared with pre-CS freezing.

To test whether PKA phosphorylation at T38 regulates multiple memory pathways, T38A mice were tested in hippocampus- and amygdala-dependent contextual fear conditioning and amygdala-dependent cued fear conditioning (Maren et al. 2013). The mice were trained with one tone–shock pairing and then tested for contextual fear memory at 24 h and 28 d after training. T38A mice had normal contextual fear memory 24 h (*t* = 0.2, two-tailed, NS) and 28 d after conditioning (*t* = 0.2, two-tailed; NS; Fig. 3A–C). Cued fear memory was also tested 48 h and 29 d after training (Fig. 3D–F). Two-way repeated-measures analysis showed that both groups of mice froze significantly more when the tone was presented but no significant difference was observed between the two genotypes 48 h (effect of genotype, *F*_(1,15)_ = 0.01, NS; genotype × time interaction, *F*_(1,15)_ = 2.2, NS) and 29 d after training (effect of genotype, *F*_(1,15)_ = 0.3, NS; genotype × time interaction, *F*_(1,15)_ = 0.2, NS). These results show that both long-term and remote contextual and cued fear memory were normal in T38A mutants.

**Figure 4.**
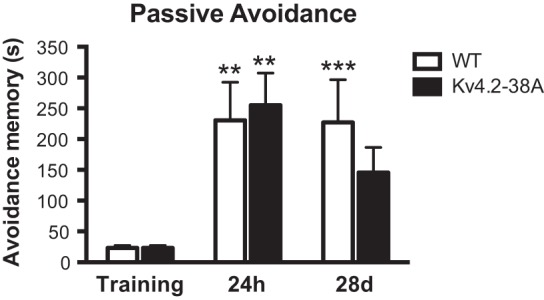
Loss of PKA phosphorylation of K_v_4.2 at threonine-38 does not affect passive avoidance memory formation. The T38A mice (*n* = 12) had normal avoidance memory 24 h as WT littermates (*n* = 9). Twenty-eight days after training only WT mice had significantly longer latencies than for training. However, at this time the latency does not differ between genotypes. Mean ± SEM. (**)*P* < 0.01; (***)*P* < 0.001 compared with training latency.

Finally, we used the passive avoidance task, which exploits a preference of mice for the dark compartment of a light–dark box, in which the animal receives a single foot shock during the training session. Memory consolidation in the passive avoidance task requires both the amygdala (Liang et al. 1982) and hippocampus (Taubenfeld et al. 2001). WT mice and T38A mutants were tested 24 h and 28 d after training (Fig. 4). Two-way repeated-measure analysis showed that both WT mice and T38A mutants formed a memory (effect of time, *F*_(2,38)_ = 17.4, *P* < 0.001) and that there was no significant difference between the genotypes (effect of genotype, *F*_(1,19)_ = 0.17, NS; genotype × time interaction, *F*_(2,38)_ = 1.0, NS). Post hoc analysis showed that WT mice had significantly longer 24-h and 28-d latencies (*P* < 0.01), whereas for the T38A mice only the 24-h latency was different from training (*P* < 0.001). However, a multiple comparisons test revealed there was no difference between the genotypes at 24 h and 28 d after training. Taken together, this indicates that T38A mice avoided the dark chamber to a similar extent as their WT littermates.

### Loss of PKA phosphorylation of SK1 at serine-105 impairs passive avoidance memory formation, but does not affect memory after fear conditioning

**Figure 5.**
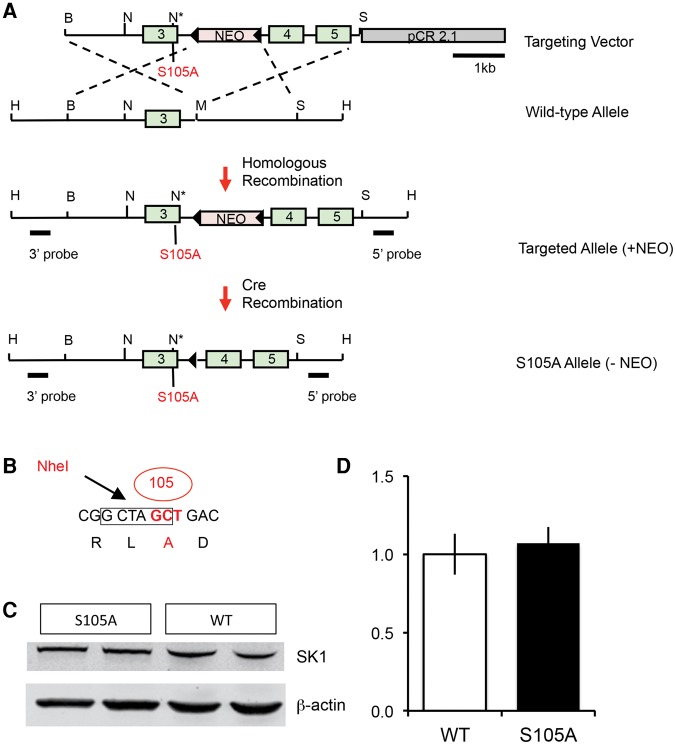
Generation of mice with a S105A point mutation in the SK1 gene. This mutation prevents PKA phosphorylation of SK1 at serine-105. (*A*,*B*) The S105 encoding sequence of the K_v_4.2 gene was mutated to an alanine-coding sequence, resulting also in the introduction of a diagnostic NheI site for detection of the point mutation. The S105A point mutation was introduced into the SK1 gene in ES cells and the floxed NEO gene was removed by Cre recombination. (B) BamHI; (H) HindIII; (N) NheI; (M) MfeI; (S) SmaI. Triangles indicate loxP sites. (*C*,*D*) Western blot analysis indicated that the loxP site in the intron does not affect overall SK1 expression in hippocampus of homozygous S105A mutants. Panel *C* shows a representative result for two S105A mutants and two WT littermates. Panel *D* shows the result of quantification of SK1 expression normalized to β-actin levels (mean ± SEM; *n* = 3 per genotype; *t*-test: *P* = 0.70).

**Figure 6.**
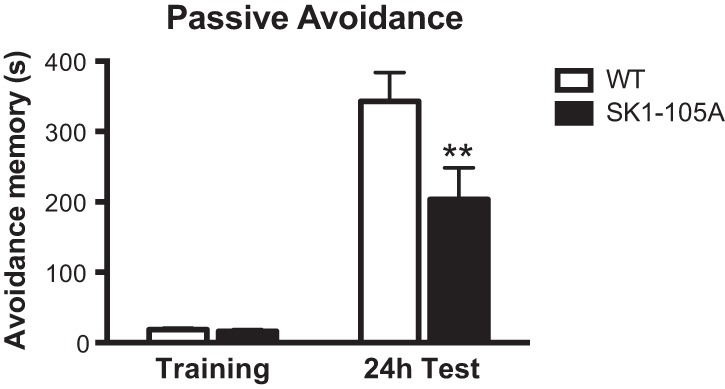
Loss of PKA phosphorylation of SK1 at serine-105 impairs passive avoidance memory formation. Twenty-four hours after training in passive avoidance the S105A mutants (*n* = 17) had impaired memory in comparison with WT littermates (*n* = 18). Mean ± SEM. (**)*P* < 0.01 compared with WT avoidance memory.

**Figure 7.**
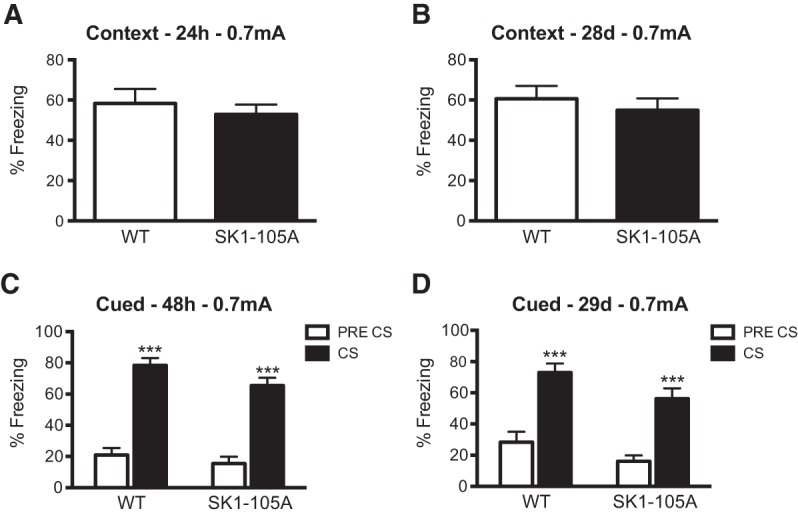
Loss of PKA phosphorylation of SK1 at serine-105 does not affect contextual and cued fear memory formation. (*A*) Mice were trained in fear conditioning with one tone/shock pairing (0.7 mA, 80 dB). Contextual fear memory was normal in S105A mice (*n* = 16) 24 h and 28 d (*B*) after training as freezing levels did not differ from WT mice (*n* = 15). (*C*) SK1 mice had normal cued fear memory when tested 48 h after training. (*D*) Twenty-nine days after training the SK1 mutants showed a subtle impairment in cued fear memory. Mean ± SEM. (***)*P* < 0.001 compared with pre-CS freezing.

To test further the notion that phosphorylation of K^+^ channels impacts on distinct cellular pathways of memory formation, we generated another targeted point mutation in the gene encoding the calcium-activated K^+^ channel subunit SK1 (Fig. 5). Since PKA modulates the AHP (Pedarzani et al. 1998) and because the AHP may depend on SK1 according to the metabolic state of the cell (Andres 2012), we inactivated the sole high-probability PKA site of SK1 at serine-105 (S105). This site is conserved between zebrafish and Fugu, as well as chimp, mouse, and human (not shown). SK1^S105A/S105A^ (S105A) mice were tested for memory formation in the passive avoidance task (Fig. 6). Two-way repeated-measure analysis showed that there was a significant interaction between genotype and time (*F*_(1,32)_ = 4.97; *P* < 0.05). Bonferroni's multiple comparisons test showed that the S105A mutants did not differ from WT mice during training, but they were impaired 24 h after training since they entered the dark compartment with shorter delay (WT, 343 sec ± 41 sec; S105A, 204 sec ± 45 sec; *P* < 0.01). This impairment in the passive avoidance task is not caused by an altered fear response in S105A mutants, as contextual and cued fear conditioning were both unaffected (Fig. 7). The mice were then trained with a single tone–shock pairing and tested for contextual fear memory at 24 h and 28 d after training. Contextual fear memory was unimpaired in S105A mutants at both 24 h (*F*_(1,29)_ = 0.40, NS) and 28 d after training (*F*_(1,29)_ = 0.44, NS; Fig. 7A,B). Cued fear memory was unimpaired 48 h after training (two-way ANOVA; effect of genotype, *F*_(1,29)_ = 0.95, NS; genotype × time interaction, *F*_(1,29)_ = 2.84, NS; Fig. 7C), with a borderline impairment 29 d after training (two-way ANOVA; effect of genotype, *F*_(1,29)_ = 4.34, *P* < 0.05; Fig. 7D).

### Loss of PKA phosphorylation of SK1 at serine-105 does not affect spatial memory

**Figure 8.**
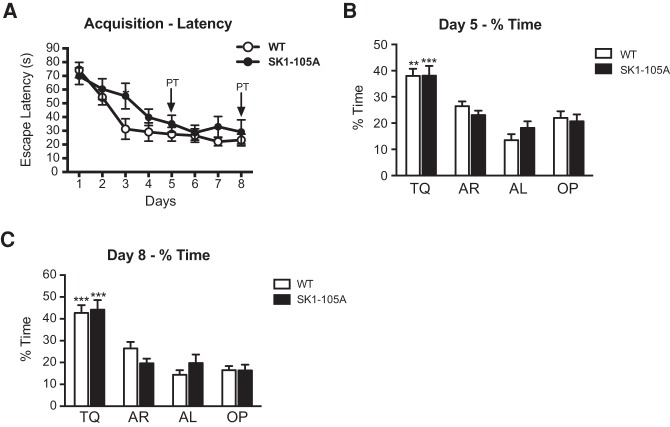
Loss of PKA phosphorylation of SK1 at serine-105 does not affect spatial memory formation in the Morris water maze. (*A*) Performance of S105A mutants (*n* = 12) and WT littermates (*n* = 13) did not differ during training. (*B*,*C*) Both genotypes searched equally selective for TQ during the probe trials on days 5 and 8. The groups did not differ in average swim speed during the probe trials on day 5 (*P* = 0.99) and day 8 (*P* = 0.71), suggesting that swimming ability and motivation were unaffected in the S105 mutants. Mean ± SEM. (**)*P* < 0.01; (***)*P* < 0.001 compared with all other quadrants.

The S105A mutants were studied in the hidden-platform version of the Morris water maze to assess whether loss of phosphorylation of SK1 at S105 affects spatial reference memory formation (Fig. 8). There was no difference in ability to locate the platform position during training (Fig. 8A), and the S105A mutants searched like WT littermates for the missing platform during probe trials on days 5 and 8 (Fig. 8B,C). Additionally, after hidden-platform training both genotypes efficiently located a visible platform (WT: 14.8 ± 7.0 sec; S105 mutants: 15.5 ± 7.1 sec; *t* = 0.065; NS). Thus, spatial reference memory formation in the water maze was spared in the S105A mutants, and the effect of altered phosphorylation at the single PKA substrate residue was confined to the impairment of passive avoidance memory.

## Discussion

We have directly tested the long-standing hypothesis that K^+^ channel phosphorylation regulates memory formation, by specifically inactivating a single PKA phosphorylation site in two different K^+^ channel subunits. In mice we inactivated the PKA phosphorylation site at threonine-38 of K_v_4.2. Loss of this particular phosphorylation site enhances spatial reference memory for several weeks, but it does not impact on other types of memory. Additionally, we inactivated the unique high-probability PKA site of SK1 at S105. Loss of this phosphorylation specifically impairs memory after passive avoidance training. Thus, in accordance with the high diversity of K^+^ channels, which enables a sophisticated fine-tuning of neuronal excitability (Pongs 2008; Jan and Jan 2012), inactivation of a single phosphorylation site in two distinct K^+^ channel subunits regulates a specific type of memory. Together, with earlier findings that the recruitment of neurons to memory circuits depends on neuronal excitability (Zhou et al. 2009; Yiu et al. 2014) this suggests that memory allocation is regulated by K^+^ channel phosphorylation at dedicated residues.

### The role of PKA phosphorylation of K_v_4.2 at threonine-38 in spatial memory formation

PKA phosphorylates K_v_4.2 at both T38 and S552 (Anderson et al. 2000). Phosphorylation at T38 occurs predominantly in the stratum lacunosum moleculare SLM in hippocampal area CA1 (Varga et al. 2000) and alters neither the biophysical properties of K_v_4.2 nor the channel's interaction with auxiliary KChip subunits (Schrader et al. 2002). Therefore, it was proposed that phosphorylation at T38 regulates the subcellular distribution of K_v_4.2 (Schrader et al. 2002). Axonal projections directly from entorhinal cortex layer 3 terminate on spines in the SLM, the so-called temporo-ammonic (TA) input (Desmond et al. 1994). Lesioning of the TA input of normal mice shortly after water maze training impairs 28-d spatial memory, suggesting that the TA input is required for consolidation of this long-term memory (Remondes and Schuman 2004). Interestingly, the effect of the TA lesion is opposite to the improved 28-d spatial memory phenotype of the T38A mice, suggesting that the TA input in mutant mice is enhanced by altered phosphorylation due either to synaptic changes or alterations in dendritic excitability. Future studies are needed to investigate this idea.

It is informative to compare the spatial memory phenotype of T38A mice and K_v_4.2 null mutants. Spatial reference memory formation in the Morris water maze is impaired in K_v_4.2 null mutants (Lugo et al. 2012), whereas it is enhanced in our T38A mutants. Loss of K_v_4.2 leads to reduced expression of auxiliary KChip subunits in different brain regions including stratum radiatum of hippocampal area CA1 (Menegola and Trimmer 2006), as well as abolishing dendritic transient K^+^ currents (Chen et al. 2006), observations that might account for impaired spatial reference memory formation in those mice. In contrast with the loss of K_v_4.2, the T38A point mutation does not affect overall K_v_4.2 expression. Given the confinement of T38 phosphorylation to the SLM within the hippocampus (Varga et al. 2000), the impact of the T38A mutation on K_v_4.2 function might occur primarily in this layer.

Dominant-negative inhibition of PKA function in the forebrain impairs spatial memory formation (Abel et al. 1997). This result appears contrary to our finding that the subtle inactivation of a single PKA site can specifically enhance spatial reference memory. However, a blanket reduction in PKA activity impairs a broad range of processes underlying behavior (Abel and Nguyen 2008). Comparison of the two sets of data suggests that PKA can act on a range of substrates to both promote and impede memory formation, and illustrates the utility of the point mutation strategy.

### The role of PKA phosphorylation of SK1 at serine-105 in passive avoidance memory formation

We found that inactivation of the sole high-probability PKA site of SK1 at S105 impairs memory formation in the passive avoidance task. This memory phenotype is the first reported neurobiological role for SK1, which unlike SK2 and SK3 has not previously been implicated in behavior. Since the AHP current of some cell types expressing SK1 can be detected only when glucose and hence ATP is depleted (Andres 2012), it is possible that PKA phosphorylation at S105 is a way of gating SK1-containing channels. The memory impairment in S105A mutants might be caused by altered AHP modulation, although in CA1 pyramidal neurons we could not identify a change in AHP currents in these mice (RDT Taylor, J Vernon, KP Giese, and P Pedarzani, unpubl.). Thus, SK1 might modulate the AHP in other locations that are also important for passive avoidance memory formation.

The memory deficit in S105A mutants is selective for passive avoidance, whereas fear conditioning is spared. The operant component of passive avoidance makes this the more complex task, since the animal must make a decision about approaching the aversive context. A dissociation of passive avoidance and cued fear conditioning impairments has been previously reported in a null mutant mouse (Weeber et al. 2000) and is replicated in our S105A mutants. Hence, passive avoidance and fear conditioning appear to engage divergent biochemical pathways, and these can be unmasked by a point mutation of a K^+^ channel subunit. Alternatively, PKA modulation of SK1 might be confined to brain structures essential for passive avoidance memory but not fear conditioning.

### Conclusion

Phosphorylation is a physiological regulator of K^+^ channel function (Park et al. 2008). Such K^+^ channel regulation has been implicated in memory formation in invertebrates and mammals (Alkon, 1984; Giese et al. 2001; Zhang and Linden 2003; Zhou et al. 2009; Oh and Disterhoft 2014; Yiu et al. 2014), although a phosphorylation site at K^+^ channels was never directly blocked. Here, we used gene targeting to inactivate a distinct phosphorylation site in two different K^+^ channel subunits in mice and we show that this subtle manipulation affects a specific type of memory formation. In this way, we establish for the first time that the SK1 subunit contributes to the encoding of behavior, and that K^+^ channel phosphorylation at a single site can regulate memory formation.

## Materials and Methods

### Generation of K_v_4.2^T38A/T38A^ mice

HindIII-, BamHI-, and PstI-fragments containing regions surrounding exon 1 of the K_v_4.2 gene were sub-cloned from the 129/Sv mouse RPCI21 library (Osoegawa et al. 2000). A targeting construct was cloned to change the codon for threonine 38 (ACT) to alanine (GCC). A 3.95 kb NcoI/NdeI fragment including the T38A mutation and a 3.2 kb NdeI/HindIII fragment were used as homology arms. A “floxed” neo-gene was inserted into the NdeI site. A PCR strategy was used to introduce the point mutation. All PCR products and junctions in the targeting vector were sequenced to ensure that no further point mutations had been introduced. Gene targeting in R1 embryonic stem (ES) cells (Nagy et al. 1993) was identified by Southern analyses. Germline chimeras, generated by blastocyst injection, were crossed with a Cre-deleter mouse strain in the C57BL/6 background to remove the selection marker (Schwenk et al. 1995). Heterozygous mutants were intercrossed with WT littermates to transmit the T38A mutation into the germline and to remove the Cre transgene. Finally, homozygous T38A mutants and WT littermate controls were obtained from intercrosses of heterozygotes. Offspring were genotyped by PCR analysis (primers: Kv4.2seq12s, 5′-TCAGATTGGAAACAGGTCAAC-3′, Kv4.2seq13a, 5′-TACACTGCAACTGGCCTATG-3′) before behavioral testing. Mice were treated according to the UK Animals (Scientific Procedures) Act 1986.

### Generation of SK1^S105A/S105A^ mice

The deduced amino acid sequence of the mouse SK1 (KCNN1) gene, accession number BC090622, was submitted to the Netphos (http://www.cbs.dtu.dk/services/NetPhosK/), a neural network programme, which predicts the occurrence of phosphorylation sites. Serine 105 was the sole high-probability site for PKA. A 7 kb HindIII subclone of SK1 was isolated from a pPAC mouse genomic library (Osoegawa et al. 2000). By PCR mutagenesis residue serine 105 in exon 3 was converted to alanine and a diagnostic NheI site was introduced. The targeting construct was assembled from the mutant exon, homology arms derived from the pPAC subclone, and a floxed neomycin cassette (Neo). This construct was electroporated into R1 ES cells (Nagy et al. 1993). The Neo was removed by Cre mediated recombination from targeted clones identified by Southern blotting. Targeted clones were injected into C57BL/6J blastocysts prior to intra-uterine transfer. High proportion male chimeras were bred with C57BL/6J females to transmit the mutation to the F1 generation. Nonsibling heterozygotes were crossed. F2 mice were genotyped by PCR (primers: 5′-AGGAGCAGGAGGAGGAA-3′ and 5′-ACCCGTAGTTACTCCTTAGGA-3′) before behavioral testing. Mice were treated according to the UK Animals (Scientific Procedures) Act 1986.

### Western blots

Hippocampi were homogenized in cold buffer (60 mM Tris–HCl, pH 6.8 with 1% SDS, 1% Tween, and protease inhibitors). Samples were heated to 70°C for 10 min, and sheared three times with a 27G needle. The homogenate was spun at 13,000 rpm for 5 min, and the pellet discarded. Soluble protein was determined by the BCA method. Twenty micrograms was loaded onto 4%–15% Tris–HCl gradient gels (Bio-Rad) and the separated proteins were electroblotted to Immobilon PVDF membrane (Millipore). Membranes were blocked in Tris-buffered saline with 3% skimmed milk, 0.2% Tween-20, 0.5% PVP, and probed with primary antibodies diluted in 0.5× blocking buffer. Primary antibodies were anti-K_v_4.2 antibody (Santa Cruz SC-11680; diluted 1:500), anti-SK1 antibody (matching residues 28–43 of the mouse protein, gift of Dr. Guy Moss, UCL: rabbit polyclonal, 275 µg/mL; diluted 1:3000), and anti-beta-actin antibody (Santa Cruz SC-1616; diluted 1:1000). Secondary antibodies (goat anti-rabbit or donkey anti-goat: Pierce) were diluted 1:15,000 in 0.1× blocking buffer. Blots were treated with Pierce ECL plus western blotting substrate and exposed to X-ray film. Blots were stripped and reprobed with anti-actin antibody, which served as a loading control. The exposure was in the linear range.

### Morris water maze studies

Mice, 3–5 mo of age, balanced for sex were studied in a setup that has been shown to be hippocampus-dependent for male and female WT mice (Angelo et al. 2003; Antunes-Martins et al. 2007). Before training, the mice were handled 2 min daily for 5–7 d, in order to reduce their anxiety levels. Mice were acclimatized to dim light conditions for 30 min prior to testing. The pool was 1.5 m in diameter, with a platform of 0.1 m diameter positioned 0.5 cm below the surface of the water. The water was maintained at 24°C –28°C throughout the trials, and made opaque by adding nontoxic white paint so that the platform was not visible. Mice were trained with four trials per day for 8 d. Each trial started from a new position of the pool selected pseudorandomly in order to discourage nonspatial learning. The maximal trial length was 90 sec, and the intertrial interval was 60 sec. In order to assess spatial memory formation probe trials were given at the end of training on day 8. To test for remote spatial reference memory a probe trial was given 28 d after the completion of training. For the probe trial the platform was removed and the mouse was introduced to the pool at the opposite quadrant and allowed to swim for 60 sec. The movement of the mice was recorded by a video tracking system (HVS Image, Hampton, UK).

### Cued and contextual fear conditioning

Mice, which were not previously studied in the passive avoidance task, were conditioned in a chamber (in the case of T38A mutants, Med Associates Inc., St. Albans, VT, USA; in the case of S105A mutants, Campden Instruments) that was placed in a soundproof box. The conditioning chamber floor was made up of stainless steel rods that were used for shock delivery. A speaker was mounted on one side of the chamber for delivery of the tone (80 dB, 3.0 kHz). Background noise was supplied to the chamber by a white noise generator positioned in the side of the soundproof box in order to camouflage any noise in the behavioral room. Prior to training the chamber was cleaned with 70% ethanol and a paper towel soaked in ethanol was placed under the grid floor. The tone testing was conducted in a novel chamber that was structurally different from the conditioning chamber. This chamber was semicircular, had a plastic floor and was lit by red light. Prior to tone test, the chamber was cleaned with a lemon-scented solution.

On the conditioning day, the mice were brought from the housing room into a holding room where they were allowed to acclimatize for 30 min before training. Mice were then placed individually in the conditioning chamber and after a 120-sec introductory period a tone (80 dB, 3.0 kHz) was presented for 30 sec, the last 2 sec of which coincided with either a 0.55 or 0.7 mA footshock. Before shock presentation none of the mice froze in the conditioning chamber. The mice were removed from the conditioning chamber 60 sec after the shock presentation and returned to their home cage. Once all mice had been trained they were returned to the housing room.

Contextual fear memory was tested 24 h after training by reexposing to the conditioning chamber for 5 min. Cued fear memory was tested 48 h after training by reexposing the mice to a novel chamber for 3 min without tone presentation, followed by 3 min with a tone presentation. In order to test for the stability of the contextual and cued fear memories the mice trained with a 0.7 mA shock were retested 28 and 29 d, respectively, after training.

A video camera was fixed inside the door of the soundproof box, which allowed the behavior to be observed and scored by an experimenter blind to the genotype of the mice. Freezing behavior (defined as complete lack of movement, except for respiration) was scored for 2 sec in every 5 sec.

### Passive avoidance studies

Mice balanced for sex, which were not previously studied for fear conditioning, were tested for avoidance memory in a light–dark box. The mice were placed individually into the lit compartment, facing away from the guillotine door of an automated passive avoidance system (Ugo Basile, Comerio, Italy). After 1 sec the door opened and the mice were allowed to enter the dark compartment. The time to cross with all 4 ft into the dark compartment was measured (=training latency). Once the mice entered the dark compartment, the door was closed automatically and the mouse received a mild footshock (0.4 mA for 2 sec). Ten seconds after the shock the mouse was removed from the apparatus and returned to the home cage. Twenty-four hours after training the mice were again placed into the lit compartment; after 1 sec the sliding door was opened and the latency (maximum 540 sec) to enter the dark compartment was scored. The mice were tested a further time 28 d after training in order to assess whether the mice had altered remote avoidance memory.

### Statistics

Data for the acquisition of the water maze, cued fear conditioning and passive avoidance were analyzed by two-way repeated-measures analysis of variance (ANOVA). Water maze probe trial data and contextual fear conditioning data were analyzed by one-way ANOVA or *t*-tests. Differences between groups were assessed by Bonferroni or Tukey's post hoc test. Non-normal data were analyzed by independent Mann–Whitney rank sum test.
